# Kinetics of C-Reactive Protein and Procalcitonin in the Early Identification of ICU-Acquired Infections in Critically Ill COVID-19 Patients

**DOI:** 10.3390/jcm12196110

**Published:** 2023-09-22

**Authors:** José Pedro Cidade, Luís Coelho, Pedro Póvoa

**Affiliations:** 1 Intensive Care Unit 4, Department of Intensive Care, São Francisco Xavier Hospital, Centro Hospitalar Lisboa Ocidental, 1449-005 Lisbon, Portugal; pedrorpovoa@gmail.com; 2 Nova Medical School, Clinical Medicine, CHRC, NOVA University of Lisbon, 1169-056 Lisbon, Portugal; luismiguelcoelho16@gmail.com; 3 Public Health Department, CDP Dr. Ribeiro Sanches, Regional Health Authority for Lisbon and Tagus Valley, 1700 Lisbon, Portugal; 4 Center for Clinical Epidemiology, Research Unit of Clinical Epidemiology, OUH Odense University Hospital, 5000 Odense, Denmark

**Keywords:** COVID-19, biomarkers, CRP, PCT, modeling analysis, ICU-acquired infections, monitoring

## Abstract

The SARS-CoV-2 infection is a cause of hypoxemic acute respiratory failure, leading to frequent intensive care unit (ICU) admission. Due to invasive organ support and immunosuppressive therapies, these patients are prone to nosocomial infections. Our aim was to assess the value of daily measurements of C-reactive protein (CRP) and Procalcitonin (PCT) in the early identification of ICU-acquired infections in COVID-19 patients. Methods: We undertook a prospective observational cohort study (12 months). All adult mechanically ventilated patients admitted for ≥72 h to ICU with COVID-19 pneumonia were divided into an infected group (*n* = 35) and a non-infected group (*n* = 83). Day 0 was considered as the day of the diagnosis of infection (infected group) and Day 10 was that of ICU stay (non-infected group). The kinetics of CRP and PCT were assessed from Day –10 to Day 10 and evaluated using a general linear model, univariate, repeated-measures analysis. Results: 118 patients (mean age 63 years, 74% males) were eligible for the analysis. The groups did not differ in patient age, gender, CRP and PCT serum levels at ICU admission. However, the infected group encompassed patients with a higher severity (SOFA score at ICU admission, *p* = 0.009) and a higher 28–day mortality (*p* < 0.001). Before D0, CRP kinetics showed a significant increase in infected patients, whereas in noninfected it remained almost unchanged (*p* < 0.001), while PCT kinetics did not appear to retain diagnostic value to predict superinfection in COVID-19 patients (*p* = 0.593). Conclusion: COVID-19 patients who developed ICU-acquired infections exhibited different biomarker kinetics before the diagnosis of those infections. Daily CRP monitoring and the recognition of the CRP kinetics could be useful in the prediction of ICU-acquired infections.

## 1. Introduction

The emergence of the SARS-CoV-2 infection presented a novel and significant clinical challenge, imposing a substantial burden on healthcare systems worldwide [1,2,3]. This unique and complex form of hypoxemic acute respiratory failure is frequently accompanied by multiorgan failure, requiring organ support, and, consequently, admission to an intensive care unit (ICU) [4]. Moreover, these patients are highly susceptible to ICU-acquired infections due to prolonged invasive support, long ICU stay, and finally several immunosuppressive therapies [3,5].

ICU-acquired infections are a common complication among critically ill patients, with estimated mortality rate reaching >40% [6]. However, diagnosing ICU-acquired infections relies heavily on maintaining a high clinical suspicion and conducting a comprehensive evaluation of radiological signs, laboratory results, and microbiological sampling, while facing the challenge of distinguishing between high rates of tissue colonization and actual infection [7,8,9,10]. Nonetheless, the importance of its recognition is paramount for timely prescription of empiric antimicrobial therapy.

These daily clinical challenges are further exacerbated in COVID-19 patients, characterized by a significant pro-inflammatory cytokine profile, extensive pulmonary involvement, and exceptionally high mortality rates [4,11,12,13,14]. Frequently, there is difficulty to distinguishing the clinical, laboratory and radiology manifestations of the underlying viral disease from a new ICU-acquired infection [15]. Currently, there is limited and conflicting evidence supporting the use of C-reactive protein (CRP) [12,16,17,18] or Procalcitonin (PCT) [6,11,19,20,21] for diagnosing ICU-acquired infections in COVID-19 patients, usually limited to the evaluation of their potential role in diagnosing ventilator-associated pneumonia. Moreover, published data often focus on the use of these biomarkers at ICU admission or ICU-acquired infection diagnosis, neglecting their behavior over time, kinetics, and their potential predictive value [22,23,24,25,26,27].

The objective of our study is to evaluate the value of daily measurements of CRP and PCT in early identification of ICU-acquired infections in COVID-19 patients. We hypothesize that CRP and PCT kinetics could be useful in predicting ICU-acquired infections in COVID-19 patients prior to infection diagnosis.

## 2. Materials and Methods

### 2.1. Study Cohort

A prospective observational cohort study was performed at an ICU of Centro Hospitalar Lisboa Ocidental. The study was approved by the National Ethics Committee for Clinical Research (reference REC: 2020_EO_02).

All adult mechanically ventilated patients admitted consecutively to the ICU between 1 January 2020 and 31 March 2021 were considered for the study. Patients were included if they had an admission to the ICU with a COVID-19 respiratory infection diagnosis and a length of stay of at least 72 h. COVID-19 respiratory infection was diagnosed using clinical and radiological criteria confirming pulmonary involvement with a SARS-CoV-2-positive RT-PCR test.

Within the study population, patients were stratified into two distinct groups: the infected group, comprising all patients with a documented ICU-acquired infection during their stay in the intensive care unit (ICU), and the non-infected group, which included all patients without a diagnosis of an intercurrent ICU-acquired infection during their ICU length of stay.

An ICU-acquired infection was defined as the presence of new clinical signs of sepsis, a change in baseline Sequential Organ Failure Assessment (SOFA) score of 2 points or more, and a positive culture that warranted the initiation of empiric antibiotic therapy, as determined through comprehensive clinical evaluation conducted by the attending physicians. These assessment criteria were considered only if they manifested at least 48 h after patient admission to the ICU and if the patient was not receiving antibiotics for at least 5 days before infection diagnosis. The non-infected group comprised all patients without criteria for an ICU-acquired infection.

**Figure 1:**
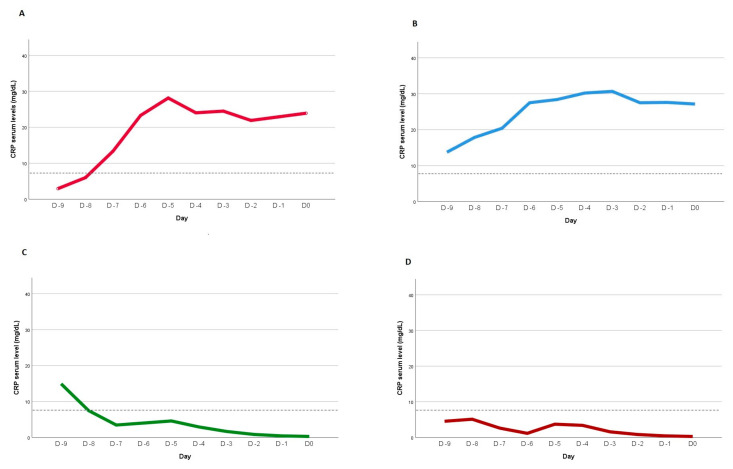
C-reactive protein (CRP) course before infection diagnosis. Four patterns of CRP course between Day −10 and Day 0 before infection diagnosis of individual patients are displayed according to a previously defined CRP cutoff value for infection diagnosis of 8.7 mg/dL [28]. See text for definition of patterns (**A**–**D**). Dashed line, CRP cutoff value for infection diagnosis.

Four CRP kinetic patterns were defined after non-linear mixed-effects modeling to the individual CRP kinetic profiles, using as a cutoff value point for infection a previously identified value of 8.7 mg/dL (Figure 1) [28]. Pattern A was defined by a Day 0 CRP serum level > 8.7 mg/dL and if, in the previous days, it was detected to be below that cutoff value at least once. Pattern B was assumed if the serum CRP level was consistently above 8.7 mg/dL until Day 0. Pattern C was defined when the serum CRP level was <8.7 mg/dL at Day 0 and was detected to be above that value at least once in the previous days. Finally, Pattern D occurred when the CRP serum level was persistently <8.7 mg/dL at all evaluations.

### 2.2. Sampling and Data Collection

To detect ICU-acquired infection, a positive microbiologic culture was considered on the following biological cultures: endotracheal aspirate, bronchoalveolar lavage or blood. For the first, a cutoff of 105 CFU/mL in culture was considered for positivity, and for the bronchoalveolar lavage, a cutoff of 104 CFU/mL was considered for positivity. For the blood samples, a microorganism documentation was used to document culture positivity. This is consistent with the recommendations provided by the Centers for Disease Control and International ERS/ESICM/ESCMID/ALAT guidelines [29,30].

Clinical data were prospectively collected from patients’ electronic health records and included demographics, comorbidities, daily laboratory values, Acute Physiology and Chronic Health Evaluation II (APACHE II) score, SOFA scores, therapies administered and data related to microbiological samples, from patients’ electronic health records. Additionally, data concerning ongoing or previous antibacterial therapy at the day of ICU-acquired infection diagnosis were also recorded to ensure patient eligibility. CRP and Procalcitonin daily values were registered in both groups. Data were stored in a pseudo-anonymized database.

The day of ICU-acquired infection diagnosis was considered to be Day 0 in the infected group. In the non-infected group, Day 10 after ICU admission was considered as Day 0 after its identification as a median day of ICU-acquired infection diagnoses in the infected group. In the non-infected group, the patients were only selected if they did not receive any antibiotic therapy in the previous 5 days before the selected Day 0. CRP and Procalcitonin daily values were registered from Day –10 to Day 10 of ICU stayed in both groups.

Biomarkers serum levels were analyzed comparing the infected and non-infected groups. All identified patterns were also compared, assessing their differences in mortality and clinical course outcomes.

### 2.3. Statistical Analysis Plan

All Gaussian distributed variables were expressed as mean (SD), and nonnormally distributed variables as median (interquartile range (IQR)). Categorical variables were expressed as numbers and percentages. To assess differences between the two main groups, the student *t* test and the Mann–Whitney U test were used for continuous variables, and the χ^2^ test was used for categorical variables.

Receiver operating characteristic curves were plotted for the maximum CRP and maximum PCT registered. The accuracy of these variables was assessed by calculating the area under the curve (AUC).

Time-dependent analysis of different variables was performed with general linear model, unmatched, univariate, repeated measures analysis using a split-plot design approach, an approach similar to that of a previous study [28].

Multivariate logistic regression model was used to assess potential variables contributing to predict the occurrence of ICU-acquired infection. The variables considered were age, gender, SOFA score at admission and maximum registered CRP and PCT serum levels, before Day 0 in ICU. They were considered to the model if statistically significant in a bivariate analysis and if they had an attributable odds ratio above 1.2. Multicollinearity was checked by computing the correlation coefficient between selected variables, and a coefficient below 0.4 was considered for exclusion of correlation. Model calibration and discrimination were accessed by the Hosmer–Lemeshow goodness-of fit test and c statistic, respectively. Results were reported as the odds ratio with the 95% confidence interval.

Non-linear mixed-effects modeling was performed on the individual CRP kinetic profiles, using as the cutoff value point for infection a previously identified value of 8.7 mg/dL. The models returned four distinct patterns, as previously described and as depicted in Figure 1. Pattern A defined patients whose CRP profiles on Day –9 fell below that cutoff and gradually increased, reaching values above the cutoff on Day 0. Conversely, Pattern B represented patients who consistently exhibited CRP values above the specified cutoff from Day –9 to Day 0. Pattern C was defined by patients whose CRP profiles were above the defined cutoff at Day –9 and reached values below the cutoff on Day 0. Patients with Pattern D consistently exhibited CRP values below the specified cutoff from Day –9 and Day 0.

Goodness of fit was assessed using the Akaike information criterion with values of 126.5 indicating the model’s suitability.

All calculations were performed in the SPSS interface (version 26.0.0.0) and R (version 4.0.3). *p*-values < 0.05 were considered statistically significant.

## 3. Results

**Figure 2:**
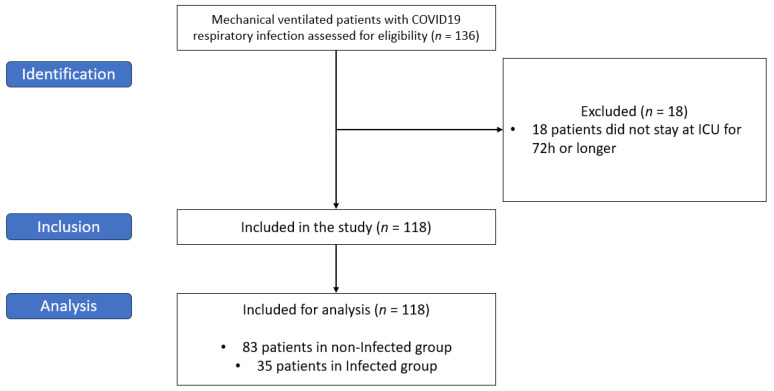
Flow chart of patient selection process using STROBE statement (STROBE stands for Strengthening the Reporting of Observational Studies in Epidemiology).

A total of 136 patients were eligible for the study. Of these patients, 18 patients did not stay at ICU for 72 h or longer and were excluded for statistical analysis. The remaining 118 patients were included, 83 (70.3%) patients in the non-infected group and 35 (29.7%) in the infected group (as depicted in Figure 2). Patient baseline demographic and primary clinical characteristics are summarized in Table 1. The cultures and microbiological identification rates are depicted in Table S1. 

The groups were not different in gender distribution, age or SAPS III at admission, although the group with ICU-acquired infections had significantly more respiratory, renal and hemodynamic organ support requirements, along with a higher SOFA score in the first 24 h after admission (SOFA score at admission, *p* < 0.001). Similarly, ICU length of stay and in-hospital length of stay were different between groups, albeit no differences in in-hospital mortality rates were identified in infected versus non-infected patients (Table 2).

**Figure 3:**
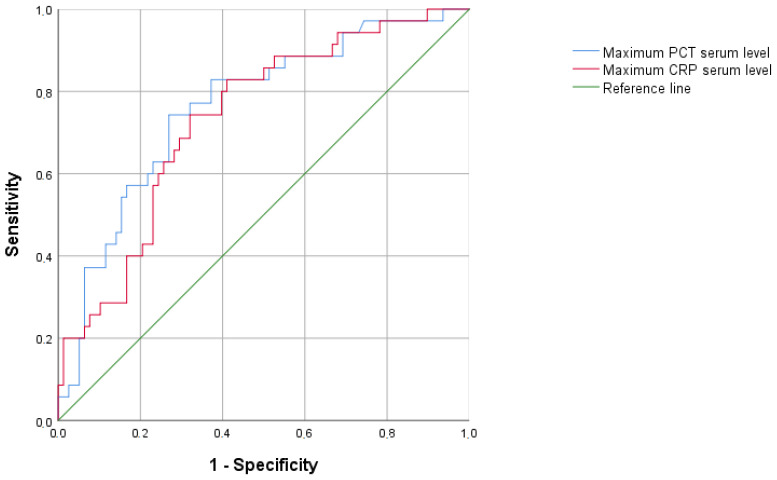
Areas under the curve (AUC) of the maximum PCT and CRP serum levels before Day 0 for prediction of ICU-acquired infection.

The median (interquartile range) of CRP and PCT were not different between non-infected and infected groups at ICU admission. However, the infected group presented significantly higher maximum values of CRP and PCT, during ICU stay, before Day 0 (Table 2). The AUC of maximum CRP and PCT as predictors of infection were 0.734 and 0.762, respectively, with an overall similar quality of the model (0.64 vs. 0.67, respectively) (Figure 3).

**Figure 4:**
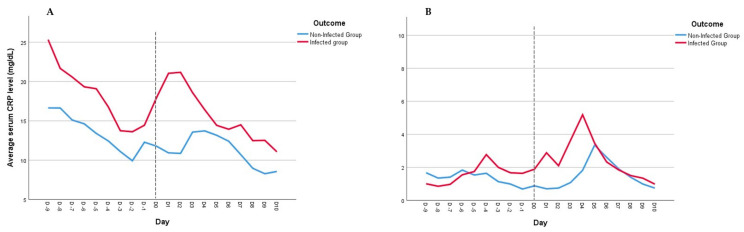
C-reactive protein (CRP) and Procalcitonin (PCT) progression before ICU-acquired infections. Time-dependent analysis of CRP (**A**) and PCT (**B**) from Day –10 to Day 0 shows a significant difference for CRP (*p* = 0.009) but not for PCT (*p* = 0.857) between patients in the infection group and the non-infection group.

Time-dependent analysis of CRP (Figure 4A) showed a significant increase in this biomarker in the 48 h before the day of infection diagnosis, whereas the CRP level in non-infected patients remained almost unchanged and steady during the days before the event of interest (*p* = 0.009). On the other hand, PCT serum levels were not different between the two groups (*p* = 0.857) (Figure 4B).

For the multivariate logistic regression model, only the SOFA score at admission, maximum CRP and PCT before Day 0 were considered in the model, and no multicollinearity was observed. Table 3 depicts the multivariable logistic regression analysis with the identification of maximum CRP registered before Day 0 as an independent predictor of ICU-acquired infection (model *n* = 89, comprehending all patients with ICU-acquired infection (*n* = 35), Hosmer–Lemeshow chi-squared 2.535 (*p* = 0.96), AUC = 0.846).

Patients were classified based on previously defined CRP kinetic patterns observed prior to Day 0. The majority of patients demonstrated CRP kinetic profiles corresponding to Pattern B (51 patients, 43.2%) and Pattern C (34 patients, 28.8%). Pattern A encompassed 9 patients (7.6%), while pattern D included 24 patients (20.4%). Key demographic and primary clinical characteristics of patients, grouped according to their respective patterns, are presented in Table S2. In terms of the distribution of CRP kinetic patterns between the non-infected and infected groups, Patterns A and B were more prevalent in the infected group, while Patterns C and D were more prominent in the non-infected group (Table 4). An analysis over time of the distinct CRP kinetics revealed statistically significant differences in these evolving patterns (*p* < 0.001). However, no discernible differences were noted in terms of clinical severity upon admission, maximum CRP levels recorded during the ICU stay, or ICU and in-hospital mortality rates among the patients.

## 4. Discussion

Our study presents compelling evidence of distinct CRP and PCT kinetics in severe COVID-19 patients with and without ICU-acquired infections. While no differences were observed between the two groups in the levels of biomarkers at ICU admission, there was a significant difference in their maximum values before the day of infection diagnosis. These findings are in accordance with previously published evidence highlighting the potential prediction value of PCT and CRP in the identification of ICU-acquired infections in COVID-19 patients [6,12,17]. However, our study advances this evaluation by emphasizing that, as single determination, these biomarkers only offer a moderately reasonable predictive value (AUC 0.73–0.76), precluding a more reasonable approach in their dynamic interpretation rather than a static value, especially considering the still unpredictable nature of the appearance of these infections. In fact, our results provide compelling evidence of the higher predictive value for infection diagnosis of CRP over PCT, using a longitudinal, time-dependent analysis of these biomarkers. The daily CRP values had a significant increase in the 48-h period preceding the diagnosis of infection, whereas non-infected patients exhibited relatively stable CRP levels. On the other hand, no significant differences were found in daily PCT levels over time between the groups.

Contrary to the findings of the studies by Farrel-Cortês et al. [11] and Richards et al. [19], our results from the multivariate logistic regression model establish CRP, rather than PCT, as an independent predictor of ICU-acquired infection with a proposed model with a more reliable prediction value, irrespective of the focus of the superimposed infection. On the other hand, our time-dependent analysis findings strongly challenge previous collected evidence based on smaller cohorts [11,19], supporting the association of this biomarker with the development of acquired infections in the ICU. Moreover, it firmly challenges previous studies proposing the use of this biomarker as a helpful tool for antibiotic withdrawal and as a stewardship tool [5].

Interestingly, our findings derived from the analysis of CRP kinetic patterns, prior to infection diagnosis, also challenge the prevailing paradigm of commonly accepted patterns as prognostic indicators for ICU-acquired infections. Within the analyzed cohort of critically ill COVID-19 patients, a persistent pro-inflammatory profile characterized by persistently elevated serum CRP levels over time was associated with a risk of developing an infection during the ICU stay, as delineated by Patterns A and B (Figure 1). Notably, patients exhibiting these CRP kinetic patterns also displayed a discernible trend towards elevated mortality rates, suggesting a potential association between sustained elevation of serum CRP and heightened rates of organ dysfunction and infection, consequently translating to poorer clinical outcomes. Concretely, almost two thirds of infected patients presented the expected A and B patterns (22 patients, 62.9%). Similarly, 54.2% of patients without ICU-acquired infection exhibit predominantly C and D patterns (Figure 1). Nevertheless, a subset of patients who acquired infections during their ICU stay also displayed an alternative pattern characterized by a decline in CRP serum levels in the days preceding infection diagnosis (Pattern C).

To our knowledge, our results show a novel clinical scenario of biomarker profiling and its predictive value in COVID-19 patients before the diagnosis of ICU-acquired infection. They further display that CRP kinetics retains an acceptable infection predictive value and should be promptly considered in conjunction with relevant clinical assessment and microbiological culture collection in septic COVID-19 patients. Therefore, our results provide evidence that continuous monitoring of CRP may have a useful role in critical care setting in these patients and has the potential to refine and compose predictor models to expedite the identification of these infections and mitigate their impact on patient survivability.

Regarding strengths, our study represents a longitudinal analysis of a reasonably large cohort of critical care COVID-19 patients, reflecting real-world clinical decision-making. Importantly, biomarker sampling was performed irrespective of clinical suspicion of ICU-acquired infection, and a high overall rate of microbiological sampling was achieved. Furthermore, none of the eligible patients in this study were treated with Interleukin-6 antagonists which could potentially bias our results.

However, we acknowledge some limitations in our study. It is retrospective in nature, and we assumed the median day of infection diagnosis as the corresponding Day 0 in the non-infected group, which may introduce determination bias due to the heterogeneous courses of COVID-19 disease over time. Furthermore, we observed an overall higher incidence of positive bacterial isolation than previously documented estimates [3], and we recognize the inherent limitations of microbiological culture methods in detecting the presence of infection. We also acknowledge that co-infection diagnosis at ICU admission in the analyzed COVID-19 patients was not considered in the kinetic pattern modeling and outcome analysis, although no difference was found in those co-infection rates between both groups (four patients (4.8%) in the non-infection group versus two patients (5.7%) in the infection group). Additionally, the independent evaluation of the attending physician was used as a key element for ICU-acquired infection diagnosis and distinguishing infection from colonization in patients with microbiological bacterial isolates. While it was not corroborated by another expert’s review, the majority of diagnoses were considered clinically relevant and treated accordingly. Nonetheless, this approach also reflects the real-world challenges faced by physicians in managing critically ill COVID-19 patients.

## 5. Conclusions

Our study presents a novel clinical scenario of biomarker profiling and its value in predicting ICU-acquired infections in COVID-19 patients. The kinetics of CRP may play a useful role in critical care settings and has the potential to refine and compose predictor models to expeditiously predict these infections, ultimately reducing their impact on patient survivability. In addition, the identification of the CRP patterns could increase our ability to identify patients with ICU-acquired infection. CRP Patterns B and C were significantly more present in COVID-19 patients with ICU-acquired infection. These findings contribute to the ongoing efforts to improve the management of ICU-acquired infections in critically ill COVID-19 patients and warrant further research to optimize the use of biomarkers in this context.

## Figures and Tables

**Table 1:** Demographic and primary clinical characteristics in the non-infected and infected groups.

	Non-Infected Group (*n* = 83 (70.3%))	Infected Group (*n* = 35 (29.7%))	*p*
Age, years (mean ± sd)	63.05 ± 14.4	63.7 ± 9.3	0.864
Gender, males (*n* (%))	61 (73.5)	26 (74.3)	0.929
Comorbidities			
Chronic Obstructive Pulmonary Disease (*n* (%))	3 (3.6)	6 (17.1)	0.08
Asthma (*n* (%))	2 (2.4)	1 (2.9)	0.88
Chronic Kidney Disease (*n* (%))	15 (18.1)	11 (31.4)	0.342
Obesity (*n* (%))	17 (20.5)	17 (48.6)	0.002
Diabetes (*n* (%))	28 (33.7)	14 (40)	0.516
Heart Failure (*n* (%))	12 (14.5)	11 (31.4)	0.03
SOFA at admission (median (IQR))	3 (2; 7)	6 (4; 9)	0.007
SAPS III at admission (median (IQR))	35 (25; 45)	38.5 (29.8; 51.3)	0.091
Mechanical Ventilation (*n* (%))	34 (41)	34 (97.1)	<0.001
Length of mechanical ventilation, days (median (IQR))	4 (0; 8)	22 (15; 29)	<0.001
Minimum paO_2_/FiO_2_ registered (mean ± sd)	151.4 ± 85.6	91.7 ± 65.5	<0.001
Vasopressor Support (*n* (%))	35 (42.2)	32 (91.4)	<0.001
Renal support therapy (*n* (%))	15 (18.1)	16 (45.7)	0.002
CRP at admission, mg/dL (median (IQR))	10 (6.2; 19.3)	11.6 (6.6; 22.8)	0.602
PCT at admission, ng/mL (median (IQR))	0.27 (0.09; 1.01)	0.58 (0.27; 1.79)	0.394

IQR denotes Interquartile range; SOFA denotes Sequential Organ Failure Assessment; SAPS denotes Simplified Acute Physiology Score; CRP denotes C-Reactive Protein; PCT denotes Procalcitonin.

**Table 2:** Primary outcomes in the Lower-value group, the Intermediate-value group and the Higher-value group.

	Non-Infected Group (*n* = 83 (70.3%))	Infected Group (*n* = 35 (29.7%))	*p*
Maximum CRP registered, mg/dL (median (IQR)) *	22.1 (14.4; 31)	32.4 (27.6; 37.4)	<0.001
Maximum PCT registered, ng/mL (median (IQR)) *	0.48 (0.2; 2.01)	5.8 (1.2; 12.6)	<0.001
ICU length of stay, days (median (IQR))	7 (4; 15)	26 (19; 37)	<0.001
In-Hospital Length of stay, days (median (IQR))	15 (12; 26.5)	36 (28; 51)	<0.001
In-Hospital mortality rate (*n* (%))	17 (20.5)	10 (28.6)	0.339

* Registered before Day 0. IQR denotes Interquartile range; CRP denotes C-Reactive Protein; PCT denotes Procalcitonin.

**Table 3:** Multivariable logistic regression model results.

	Odds Ratio	95% Confidence Interval	*p*
SOFA score at admission	1.105	0.947–1.301	0.257
Maximum registered CRP *	1.375	1.228–1.445	0.001
Maximum registered PCT *	1.009	0.876–1.023	0.671

* Registered before Day 0; SOFA denotes Sequential Organ Failure Assessment; CRP denotes C-Reactive Protein; PCT denotes Procalcitonin.

**Table 4:** Distribution of CRP kinetic patterns between non-infected and infected groups.

	Non-Infected Group (*n* = 83 (70.3%))	Infected Group (*n* = 35 (29.7%))	*p*
CRP Kinetic Patterns (*n* (%))			0.029
Pattern A	6 (7.2%)	3 (8.5%)	
Pattern B	32 (38.6%)	19 (54.3%)	
Pattern C	22 (26.5%)	12 (34.3%)	
Pattern D	23 (27.7%)	1 (2.9%)	

## Data Availability

The datasets generated and/or analyzed during the current study are not publicly available due to privacy issues, but are available from the corresponding author on reasonable request.

## Supplementary Materials

The following supporting information can be downloaded at: https://www.mdpi.com/article/10.3390/jcm12196110/s1, Table S1: Characterization of microbiological cultures (A) and Isolated microorganisms (B) in the analyzed population; Table S2: Demographic and primary clinical characteristics according to CRP kinetic patterns.
