# Supplementary material for: Kinetics of C-Reactive Protein and Procalcitonin in the Early Identification of ICU-Acquired Infections in Critically Ill COVID-19 Patients
Source: J Clin Med. 2023 Sep 22;12(19):6110. doi: 10.3390/jcm12196110 (PMC10573639; doi:10.3390/jcm12196110)
Supplement: Supplementary file 1 [file jcm-12-06110-s001.zip › jcm-2601534-supplementary.pdf]

Table S1. Characterization of microbiological cultures (A) and Isolated microorganisms (B) in the analyzed population.

A

**Culture**	***n***
Lower respiratory tract	96
Blood	156

B

**Isolated Microorganisms**	***n* (%)**
Citrobacter koseri	4
Corynebacterium striatum	1
Enterobacter cloacae	1
Enterococcus faecalis	4
Escherichia coli	1
Klebsiella oxytoca	1
Klebsiella pneumoniae	11
Neisseria meningitidis	1
Proteus mirabilis	2
Providentia stuartii	1
Pseudomonas aeroginosa	3
Serratia marcescens	1
Staphylococcus aureus	7
Staphylococcus epidermidis	7
Streptococcus agalactiae	1
Streptococcus constellatus	1
Streptococcus pneumoniae	4

Table S2. Demographic and primary clinical characteristics according to CRP kinetic patterns.

	Pattern A<br>(*n* = 3 (8.5%))	Pattern B<br>(*n* = 19 (54.3%))	Pattern C<br>(*n* = 12 (34.3%))	Pattern D<br>(*n* = 1 (2.9%))	*p*
Age, years (mean ± sd)	68.3 ± 8.4	64.5 ± 10.4	61.1 ± 8.1	67	0.602
Gender, males (*n* (%))	2 (66.7)	13 (68.4)	10 (83.3)	1 (100)	0.731
SOFA at admission (median (IQR))	3 (2.3;3.3)	5 (3.5; 8)	8 (4.3; 12.5)	1	0.061
SAPS III at admission (mean ± sd)	42.3 ± 7.5	40.6 ± 13.9	44.1 ± 13.8	25	0.419
Mechanical Ventilation (*n* (%))	3 (100)	18 (94.7)	12 (100)	1 (100)	0.833
Length of mechanical ventilation, days (mean ± sd)	28.7 ± 13.3	21.6 ± 9.9	24.5 ± 8.7	9	0.467
Minimum paO2/FiO2 registered (mean ± sd)	72.8 ± 10.6	93.9 ± 20.6	91 ± 26.1	112	0.676
Vasopressor Support (*n* (%))	3 (100)	19 (100)	10 (83.3)	1 (100)	0.003
Renal support therapy (*n* (%))	2 (66.6)	9 (47.4)	5 (41.7)	5 (100)	0.689
Maximum CRP registered, mg/dL (mean ± sd)*	29.4 ± 1.8	34.9 ± 7.3	32.5 ± 13.2	8.24	0.112
Maximum PCT registered, ng/mL (median (IQR)*	8.8 (3.5; -)	8.4 (2.9; 12.9)	1.2 (0.4; 8.7)	0.27	0.089
ICU length of stay, days (median (IQR)	32 ± 16.8	27.7 ± 11.9	28.1 ± 12.7	13	0.612
In-Hospital Length of stay, days (median (IQR))	48.3 ± 29.2	46.3 ± 29	44.8 ± 29.3	28	0.820
In-Hospital mortality rate (*n* (%))	1 (33.3%)	7 (36.8)	2 (16.7)	0 (0)	0.593

IQR denotes Interquartile range; SOFA denotes Sequential Organ Failure Assessment; SAPS denotes Simplified Acute Physiology Score; CRP denotes C-Reactive Protein; PCT denotes Procalcitonin.
*Registered before Day 0
